# MicrobiomeNet: exploring microbial associations and metabolic profiles for mechanistic insights

**DOI:** 10.1093/nar/gkae944

**Published:** 2024-10-23

**Authors:** Yao Lu, Fiona Hui, Guangyan Zhou, Jianguo Xia

**Affiliations:** Institute of Parasitology, McGill University, Quebec, Canada; Department of Microbiology and Immunology, McGill University, Quebec, Canada; Institute of Parasitology, McGill University, Quebec, Canada; Institute of Parasitology, McGill University, Quebec, Canada; Institute of Parasitology, McGill University, Quebec, Canada; Department of Microbiology and Immunology, McGill University, Quebec, Canada

## Abstract

The growing volumes of microbiome studies over the past decade have revealed a wide repertoire of microbial associations under diverse conditions. Microbes produce small molecules to interact with each other as well as to modulate their environments. Their metabolic profiles hold the key to understanding these association patterns for translational applications. Based on this concept, we developed MicrobiomeNet, a comprehensive database that integrates microbial associations with their metabolic profiles for mechanistic insights. It currently contains a total of ∼5.8 million known microbial associations, coupled with >12 400 genome-scale metabolic models (GEMs) covering ∼6000 microbial species. Users can intuitively explore microbial associations and compare their corresponding metabolic profiles. Our case studies show that MicrobiomeNet can provide mechanistic insights that are consistent with the literature. MicrobiomeNet is freely available at https://www.microbiomenet.com/.

## Graphical Abstract

**Graphical Abstract:**
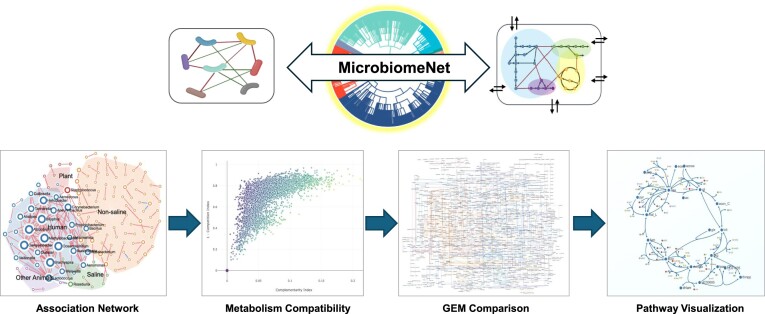

## Introduction

Microbial communities are ubiquitous in our ecosystems and play important roles in a broad set of processes such as nutrient cycles, environmental resilience, as well as host development and immunity (1,2). Recent large-scale initiatives including the Human Microbiome Project (HMP) and the Earth Microbiome Project (EMP) have catalyzed an explosion of microbiome studies carried out by different research groups (3–5). The current focus has shifted gradually from measuring community compositions towards understanding their interactions and translational applications (6–9).

Identification of co-occurrence patterns is often the first step to elucidating community interactions. Multiple statistical methods such as SparCC (10), FlashWeave (11) and SECOM (12) have been developed to address the challenges posed by sparse, heterogeneous, and zero-inflated microbiome data. These methods are routinely used in microbiome data analysis, yielding a large volume of significant associations under diverse conditions (13,14). Navigating these complex relationships for mechanistic insights remains a daunting task. Comprehensive databases equipped with easy-to-use data mining tools are urgently needed. For instance, a recently tool, mako (15), enables network-based queries to a large collection of microbial associations based on ∼60 studies from QIITA (16). However, how to obtain functional insights from these associations remains largely unaddressed.

Microbes produce small molecules or metabolites to communicate with each other and to modulate their environments. Metabolic interactions are widely recognized as important factors in community assembly and functions. For example, a well-documented interaction between *Bacteroides fragilis* and *Faecalibacterium prausnitzii* exists in human gut microbiome, where *B. fragilis* aids in the degradation of complex polysaccharides, providing simpler substrates that *F. prausnitzii* can ferment, thereby promoting gut health and maintaining microbial diversity (17).

A genome-scale metabolic model (GEM) is a network that represent the entire set of metabolic reactions in an organism, derived from its annotated genome (18). GEMs have proven to be valuable for predicting important ecological attributes, including an organism's nutritional requirement, its interaction with its host or other species (19–23). For instance, GEMs can reveal metabolites that must be acquired exogenously (also known as seed nutrients) for an organism to survive. Two species may compete if their seed nutrients overlap. On the other hand, one species can support the other species by synthesizing the seed nutrients of the latter.

Over past few years, a substantial collection of high-quality GEMs has been generated for 1000s of microbes, notably AGORA and CarveMe (19,21,24). By leveraging these resources, we developed MicrobiomeNet, a comprehensive database that allows researchers to search and explore known microbial associations and compare their underlying metabolic profiles for mechanistic insights. Its main features are described below.

## Data collection and processing

**Figure 1.**
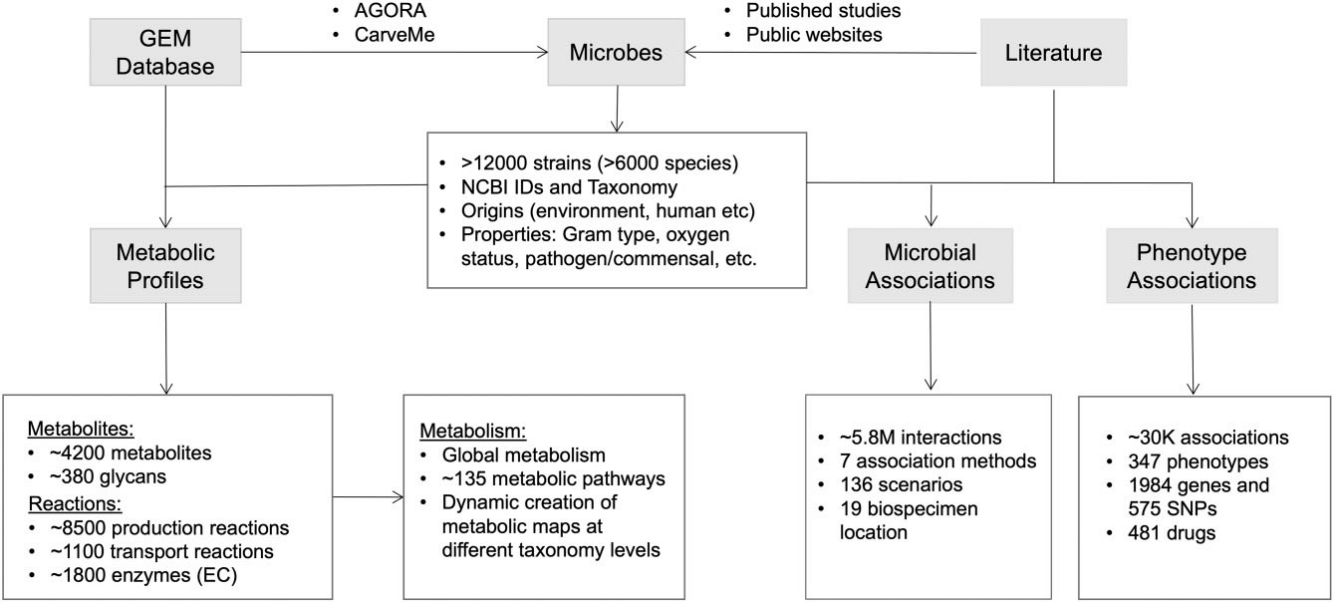
Summary of the MicrobiomeNet database content.

MicrobiomeNet currently contains three main data types. First, it hosts over 12 400 GEMs covering over 6000 microbial species, primarily within the domains of bacteria and archaea. The metabolic maps were further deconstructed into their components (metabolites, reactions, and pathways) to facilitate searching and computing. Second, it includes ∼5.8 million microbial associations. These associations were categorized into 139 scenarios and were assigned to 19 biospecimen locations according to the EMP ontology level 3. Finally, MicrobiomeNet contains ∼30 000 microbial signatures based on their associations with various phenotypes, host genetics, and drugs. Most of these associations were derived from the human studies. Figure 1 summarized the detailed content captured in MicrobiomeNet.

### Collection and curation of GEMs

The original GEM files (.mat or .xml) were downloaded from the AGORA and CarveMe websites, respectively (21,24). These model files were parsed to extract the information on taxonomy, metabolites, reactions, and pathways. The taxonomy lineages of these GEMs were updated based on the NCBI taxonomy database (25). Key microbial properties such as Gram staining, oxygen preference, and pathogenicity were retrieved using the BacDive API (26), followed by manual verification from public databases. The biospecimen origins of the GEMs were primarily determined through literature reviews. Missing or outdated information regarding metabolites and reactions were updated using KEGG, HMDB, ModelSeed, BIGG, MetaCyc and MetaNetX (27–32). Metabolic pathways were defined during the reconstruction process based on the subsystems approach (33). The refined information was organized into tables for browsing and searching. Based on the reactions presented in each microbe, we generated their metabolic pathways and global metabolic networks.

### Collection and curation of microbial association data

The microbial co-occurrence patterns were manually compiled from 76 published studies covering different taxonomy levels from phylum to species. The statistical associations were mainly generated using Pearson, Spearman, SparCC and FlashWeave in the original papers with an adjusted *P*-value cutoff 0.05. We maintained the original significance thresholds used for each study. These studies were further organized into three levels based on the Earth Microbiome Project Ontology (EMPO) (5), and taxonomic names were further updated based on the NCBI taxonomy database (25). For duplicated interactions detected between the same pair of taxa, their average values are used for visualization. Finally, the curated microbial associations were parsed into a node table and an edge table for searching and network creation.

### Collection and curation of other microbial signatures

In addition to microbial co-occurrence patterns, human microbiome studies have also yielded many signatures associated with different phenotypes (such as various diseases), host genetics and drugs. These associations provide important contexts and clues for understanding microbial functions (34). We collected these taxon sets from both literature as well as public resources such as MicrobiomeAnalyst, gutMDisorder and GIMICA (34–38). The patterns of increased or decreased associations were also curated from the original studies, together with their cohort information when available. All taxa were further updated to the NCBI taxonomy names. The associations for each feature type were compiled and organized into separate tables for browsing and searching.

### Computing microbial neighbourhood map and metabolism compatibility map

To provide an overview of the potential relationships between microbial co-occurrence patterns and their taxonomic or metabolic profiles, MicrobiomeNet computes *Neighbourhood Map* and *Metabolism Compatibility Map* based on the curated GEMs.

**Figure 2.**
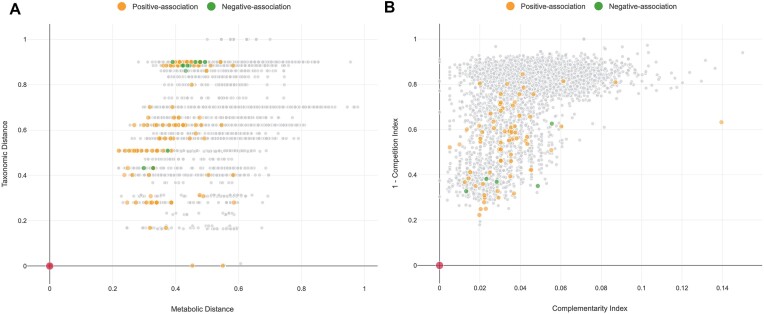
(**A**) *Neighbourhood Map* and (**B**) *Metabolism Compatibility Map* for a query microbe *Faecalibacterium prausnitzii*,represented by the red point at the origin. Each data point represents an entry in the MicrobiomeNet database. The colored points represent taxa reported to be associated with it.

As shown in Figure 2A, a*Neighbourhood Map* integrates metabolic distance (*x*-axis) and taxonomic distances (*y*-axis) for all taxa covered in MicrobiomeNet for a given microbe of interest. Metabolic distances were computed based on the presence or absence of specific reactions in the given taxa. Taxonomic distances were calculated according to the NCBI taxonomy using the *ape* R package (39).

MicrobiomeNet can also calculate the competition and complementarity indexes for any pair of microbes. Firstly, seed metabolites are identified which are defined as the minimal group of external compounds required by a specific organism to produce all other compounds in the metabolism. The Kosaraju algorithm (40) was used to break down the metabolic network into strongly connected components (SCC). Seed metabolites were selected from those SCCs that have no incoming edges but at least one outgoing edge (41). As suggested by the original publication, we further restricted the seed metabolites to GEMs with transport or exchange reactions identified, and SCCs containing at least five metabolites. The competition index describes the similarity of the nutrition requirements between two microbes, denoted by the proportion of shared seed metabolites between microbe A and B. Similarly, the complementarity index from microbe A to B is calculated as the proportion of compounds that can be produce by microbe A but absent in its seed metabolites while present in the seed set of microbe B (22). Both indexes were further normalized by their respective seed numbers. For any query microbe, MicrobiomeNet can generate a *Metabolism Compatibility Map* based on the mean pair-wise complementarity and competition indexes between the microbe and all other microbes (Figure 2B).

## Database interface and main featuers

MicrobiomeNet provides a user-friendly web interface equipped with a suite of functions for browsing, searching and visualization. These features allow users to uncover valuable insights and foster a deeper understanding of microbial ecosystems.

### Browsing metabolic profiles of individual microbes

The overall summary statistics of the database content are shown on the home page. Users can browse the content using the top menu items. Click each menu (Microbes, Metabolites, Reactions or Pathways) will lead to the corresponding table view with links to more details. The Microbe table displays the taxonomy and multiple properties, such as the biospecimen source, Gram stain, oxygen tolerance and pathogenicity. Their metabolic profiles include the number of metabolites, reactions, and pathways. Other tables describe the chemical properties of metabolites, formula of reactions, and pathway contents as well as their related microbes. The frequencies of each metabolite and reaction among all GEMs in the database are also presented. All tables are sortable, searchable and interconnected, allowing for intuitive navigation and seamless exploration of data.

### Searching and exploring microbial associations

The search option on home page is the main entry point to the underlying database. Users can search across six distinct categories: microbes, metabolites, enzymes, phenotypes, genes/SNPs, or drugs. The autocomplete function allows efficient search of the information stored in the database. The search results will be presented as a table hyperlinked to association networks, GEMs, pathways and other graphical summaries.

Users can search a microbe to identify its known associations. From the result table, users can use the corresponding hyperlinks to visualize its metabolic profiles (metabolism and pathways), the association networks, as well as the corresponding *Neighbourhood Map* and *Metabolism Compatibility Map*. Similarly, users can search a metabolite or enzyme to identify the microbes capable of metabolizing a given metabolite or catalyzing a reaction, respectively. The results also contain hyperlinks to the related pathways and GEMs. MicrobiomeNet supports enzyme name searches at four different levels as defined by the Enzyme Commission (EC). The search results for main classes or subclasses include all enzymes in the database that fall into the specified category. Users can also enter a phenotype (i.e. a disease), gene, SNP or drug name to search for its associated microbial signatures. The results are presented as a table with columns detailing the relationship with the microbe, host, cohort, as well as reference publications.

### Visual exploration of microbial association networks and metabolic profiles

Microbial association networks can be accessed via the search function for a microbe or the ‘Visualize’ function at the home page (under the ‘Microbial Association Networks’ section). The default view presents a summary of all current microbial associations, with color-coding based on different sample sources according to the EMPO level 1. Users can perform further filtering by study contexts, taxonomy levels, computational methods, or significance thresholds. Clicking on a node or edge will show more details with links to their metabolic profiles.

The metabolism of a microbe can be visualized at global level (GEM view) or pathway level. At both levels, nodes represent metabolites and edges represent reactions. The default GEM view includes all pathways except for a few very large pathways, such as *Fatty Acid Biosynthesis, Glycerophospholipid Metabolism* and *Drug Metabolism*, which are visualized separately for rendering efficiency. Users can color the edges by microbes to visually compare the metabolisms of selected microbes. The GEM view can also display seed metabolites as well as to compute competition and complementarity indexes for selected taxa of interest. Cofactors are specifically supported in the pathway view.

### Implementation of the database and web interface

MicrobiomeNet database was implemented based on DuckDB (https://duckdb.org/) for data storage and fast search operation. The web interface was implemented using the PrimeFaces component library (version 13.0.10). The interactive networks were implemented utilizing sigma.js (https://www.sigmajs.org/) and d3.js (https://d3js.org/) libraries. Statistical functions and static figures were implemented using R (version 4.4.1, https://www.r-project.org/).

## Use cases

As previously described, *F. prausnitzii* is one of the most abundant commensals in human gut. It has been reported to be associated with *B. fragilis* and is involved in several diseases such as inflammatory bowel disease (IBD). Here, we use *F. prausnitzii* as an example query microbe to showcase the main features of MicrobiomeNet.

**Figure 3.**
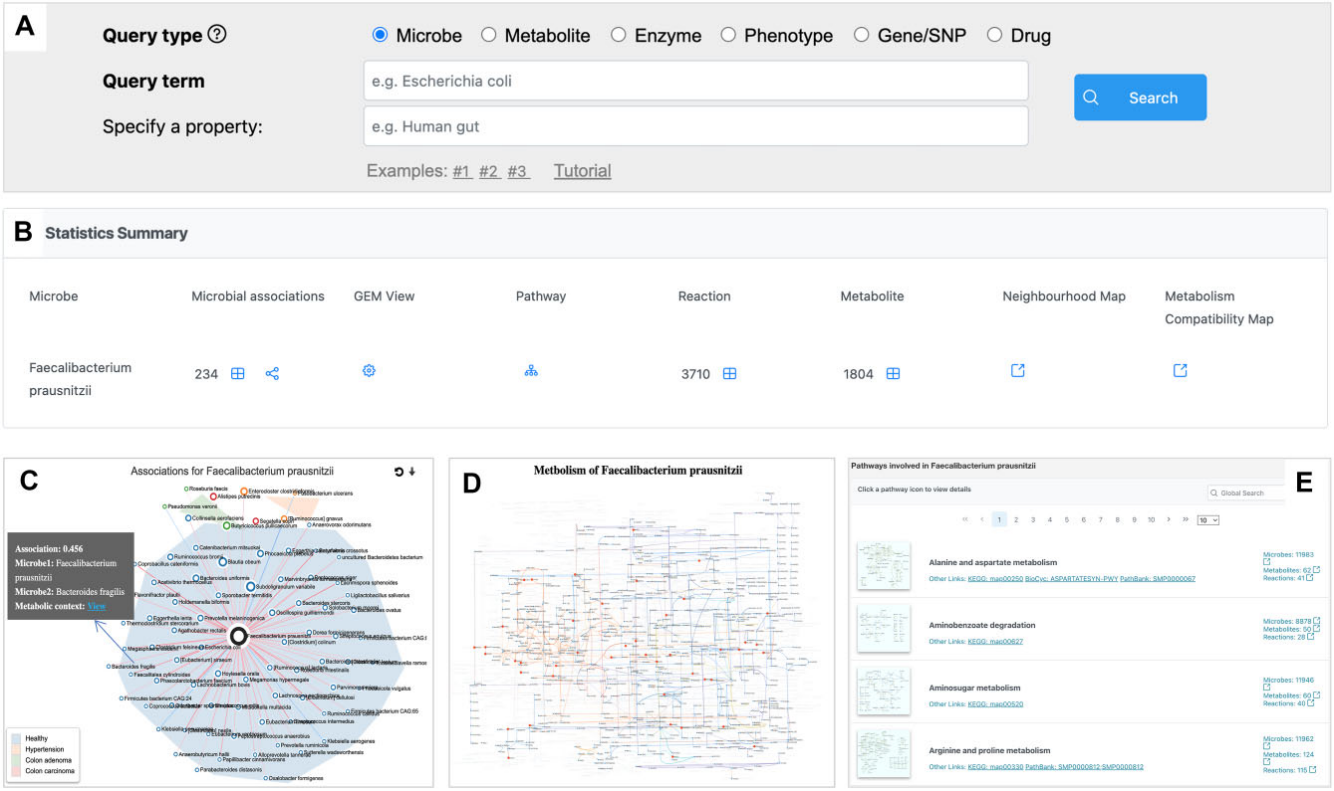
Screenshot illustrations of the procedures for querying MicrobiomeNet for microbe *F. prausnitzii*. (**A**) search bar for input, (**B**) summary of the search result, (**C**) microbial association network, (**D**) global metabolic network and (**E**) pathway table.

Users can start with the search function on the homepage by pasting the name of the microbe (*Faecalibacterium prausnitzii*) or typing its name assisted by the autocomplete feature (Figure 3A). The example described here can also be performed using the first example. After clicking the search button, the results page displays summary statistics regarding the known microbial associations, metabolites, reactions, and pathways of the query microbe. Figure 3B shows the table view of the 237 microbial associations, 3710 reactions and 1804 metabolites, respectively found for *F. prausnitzii* in MicrobiomeNet database. Users can click the corresponding icons to view a more detailed table information or visualize the association networks. The table includes the computational methods used and the weights of the associations. Users can also filter microbial associations based on context. Click the network icon to view the association network centered on *F. prausnitzii*, with all the associations that found to occur in the human gut across different biological contexts (Figure 3C). Figure 3D shows the global metabolic network of *F. prausnitzii* with seed metabolites highlighted. Figure 3E display the metabolic pathways in *F. prausnitzii*. Users can navigate to the corresponding pathway page by clicking the icon for each pathway. The corresponding *Neighborhood Map* highlighted with all microbes associated with *F. prausnitzii* is shown in Figure 2A. In this case, most taxa that are negatively correlated with *F. prausnitzii* fall within a narrow range of taxonomic distance at around 0.9, suggesting distinct ecological niches between these microbes and *F. prausnitzii*. These taxa are also positioned within a metabolic distance range of approximately 0.35–0.5, indicating potential competition with *F. prausnitzii*. Microbes positively associated with *F. prausnitzii* exhibit a wide range of taxonomic distances, while their metabolic distances are consistently <0.6, highlighting the impact of metabolic compatibility in shaping microbial interactions. The *Metabolism Compatibility Map* (Figure 2B) shows the complementarity (*x*-axis) and 1−competition (*y*-axis) indexes between *F. prausnitzii* and other species. The negatively associated taxa exhibit a relatively higher average competition index compared to the positively correlated taxa, potentially explaining the observed negative association.

**Figure 4.**
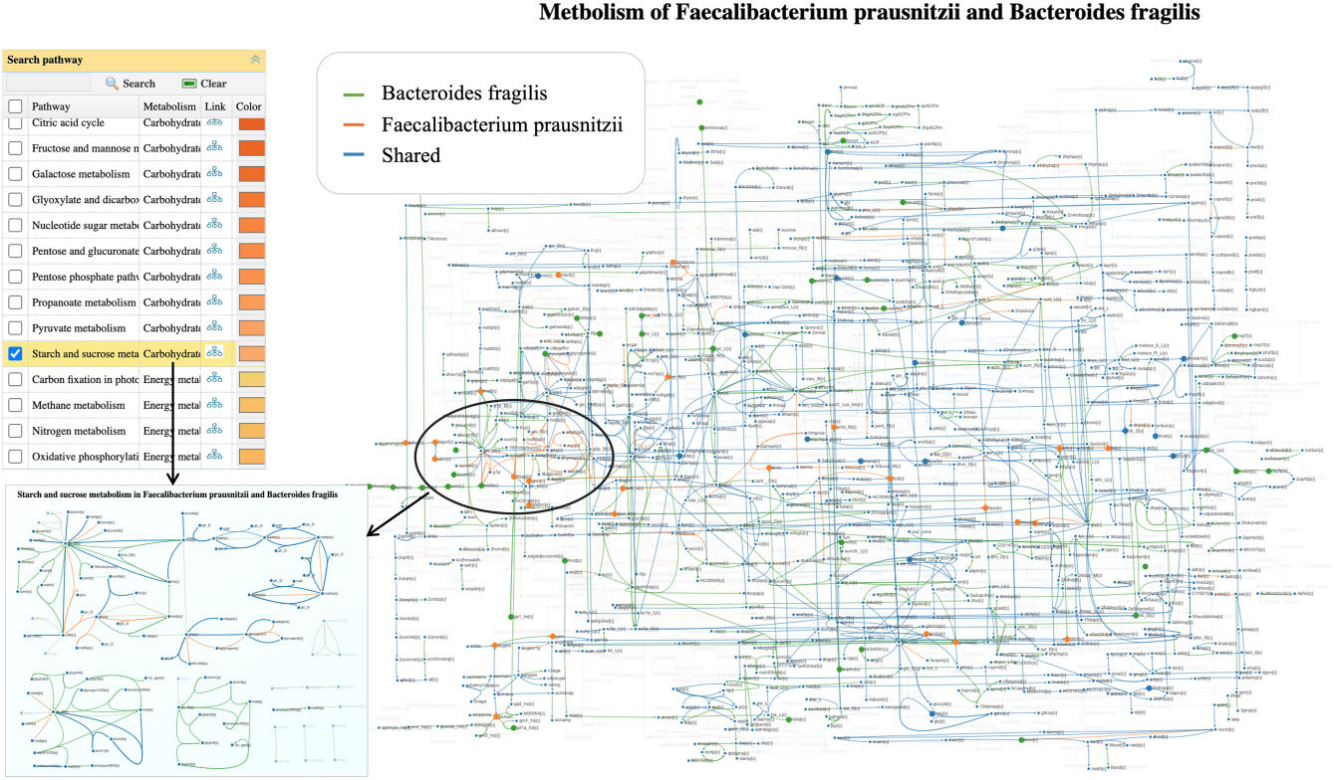
Visual exploration of the potential metabolic interactions between *F. prausnitzii* and *B. fragilis*. The figure compares the metabolic profiles of the two species with the seed metabolites highlighted. The pathway view can be accessed by clicking the icon in the top left panel. The *starch and sucrose metabolism* pathway is shown on the bottom left corner to illustrate significant distinctions between the two microbes.

Based on the above observation, we focus on the association of *B. fragilis* with *F. prausnitzii*, which is found to be present in the healthy human gut. To explore their potential metabolic interactions, click the edge between these two microbes in the association network (Figure 3C). In the pop-up panel, click the ‘View’ to visually compare their metabolic networks (Figure 4). In the GEM view, users can highlight a pathway in the current map or navigate to the pathway view by clicking the icon in the left panel. Figure 4 shows the *Starch and sucrose metabolism* which has notable differences between the two microbes. To further understand the positive correlation reported, we calculated the complementarity index. *B. fragilis* ranks among the top 5% for complementarity index scores when compared to the index between *F. prausnitzii* and other species. This finding is consistent with the previous research, which shows that *B. fragilis* digests complex polysaccharides into simpler sugars, such as glucose, which supports the growth of *F. prausnitzii*. In turn, *F. prausnitzii* produces butyrate as a byproduct, which is essential for maintaining the health of the colonic epithelium and preventing inflammation.

MicrobiomeNet also allows users to search features other than microbes. Here we show an example query with a metabolite, *butyrate*. The example can be achieved by using the first example for metabolite input. The result table displays all microbes capable of metabolizing this metabolite. More than half microbes in GEM are involved, with about 1000 of them found in the human gut, including nine strains from the species *F. prausnitzii*. Butyrate is produced through *Butanoate metabolism*, though the metabolic pathways for butyrate differ among strains. By click the pathway hyperlink, users can access detailed information on how each strain metabolizes butyrate.

## Conclusion and discussion

The wide applications of next-generation sequencing and high-resolution mass spectrometry techniques have greatly fueled microbiome studies, enabling the identification of complex co-occurrence patterns under diverse experimental conditions and study contexts. Understanding the microbial community functions and their metabolic interactions are the natural next step.

Several web-based databases have been developed to archive results from microbiome studies, such as the Microbe and Disease Phenotype Association Database (MicroPhenoDB) (42) and the Human Microbe-Disease Association Database (HMDAD) (43). However, curating the association patterns represents only the first step towards understanding the mechanisms of interactions. To address this limitation, the Human Microbial Metabolome Database (MiMeDB) (44) was developed to link microbes, microbial-derived metabolites within the context of human microbiome and metabolome. MiMeDB represents a significant step for mechanistic understanding of microbiome. However, it currently supports a limited range of microbes as it is dedicated to the human microbiome and lacks support for metabolic networks, which are crucial to help understand how microbes consume, transform, and produce metabolites, driving interactions within the microbial community and with the host.

We developed MicrobiomeNet as a comprehensive database that allows researchers to easily search and visually explore existing knowledge of microbial associations and their underlying metabolic profiles. To the best of our knowledge, MicrobiomeNet is currently the largest public database of microbial associations curated from microbiome studies, and the most comprehensive web-based platform that supports interactive visualization of GEMs. It aims to address the gap by correlating the observed patterns with their metabolic profiles. As demonstrated in our case studies, users can search a microbe of interest to identify its known associations, examine their overall similarities through the *Neighborhood Map* or *Metabolism Compatibility Map*, and finally delve into the metabolic profiles of key interaction partners for mechanistic insights.

Understanding the metabolic functions of the microbiome in isolation is insufficient to fully explain the complex interactions between microbes and their hosts. Integrating host metabolism data will be crucial in uncovering the intricate host-microbe crosstalk, offering a more holistic view of these interactions. In addition, incorporating the concept of pan-model to mimic the complex microbial communities will provide deeper insights into microbial ecosystem and enhance our ability to predict metabolic interactions within diverse microbial populations (45,46). These new features will be implemented in the future releases of MicrobiomeNet.

## Data Availability

The resources of MicrobiomeNet are freely accessible and downloadable at https://www.microbiomenet.com/.
